# Endoscopic observation of the palisade vessels in Killian–Jamieson diverticulum was useful for diagnosis and surgical treatment: a case report

**DOI:** 10.1186/s40792-020-00949-z

**Published:** 2020-08-03

**Authors:** Yasunori Kurahashi, Yudai Hojo, Tatsuro Nakamura, Tsutomu Kumamoto, Yasutaka Nakanishi, Yoshinori Ishida, Hisashi Shinohara

**Affiliations:** Department of Surgery, Upper Gastrointestinal Division, Hyogo College of Medicine, 1-1 Mukogawa-Cho, Nishinomiya City, Hyogo 663-8501 Japan

**Keywords:** Pharyngoesophageal diverticulum, Killian–Jamieson diverticulum, Upper esophageal sphincter (UES), Palisade vessels, Cricopharyngeal myotomy

## Abstract

**Background:**

Killian–Jamieson diverticulum is a rare pharyngoesophageal diverticulum that arises below the cricopharyngeus muscle. Unlike the most common Zenker’s diverticulum, which requires cricopharyngeal and esophageal myotomy, diverticulectomy is sufficient for surgical treatment of Killian–Jamieson diverticulum. Thus, accurate preoperative diagnosis is indispensable for avoiding unnecessarily invasive surgery. Here, we report a case of Killian–Jamieson diverticulum in which endoscopic observation of the palisade vessels was useful for diagnosis and intraoperative endoscopy was effective in guiding surgical resection.

**Case presentation:**

A 65-year-old woman complained of pharyngeal discomfort and increased coughing and was referred to our hospital with a diagnosis of a pharyngoesophageal diverticulum. Contrast esophagography and cervical computed tomography revealed a diverticulum measuring 3 cm in diameter on the left side of the cervix. The diverticulum was identified by endoscopy just below the palisade vessels, which represents the level of the upper esophageal sphincter, and was diagnosed as Killian–Jamieson diverticulum. She underwent diverticulectomy without cricopharyngeal and esophageal myotomy. After exposing the diverticulum under light from the endoscope and washing out the food residue inside endoscopically, the diverticulum was resected using the endoscope as a bougie so as not to narrow the esophagus. The postoperative course was uneventful, and she remains asymptomatic without recurrence or stenosis at 6 months after surgery.

**Conclusions:**

Endoscopic observation of the palisade vessels in addition to esophagography can help diagnose Killian–Jamieson diverticulum and determine the optimal surgical procedure. Diverticulectomy can be performed intentionally and safely with the aid of intraoperative endoscopy.

## Introduction

Surgical treatment of the pharyngoesophageal diverticulum, known as Zenker’s diverticulum (ZD), which arises above the cricopharyngeus muscle, requires cricopharyngeal and esophageal myotomy in addition to diverticulectomy [1–3]. Killian–Jamieson diverticulum (KJD), on the other hand, which arises below the cricopharyngeus muscle, does not require myotomy [2, 4]. Reliably distinguishing between the two types of diverticulum is therefore important when planning a surgery. Diagnosis is usually made by contrast esophagography, but it sometimes cannot be confirmed.

Here, we present a case in which endoscopy proved useful diagnostically in the confirmation of KJD, by identifying the level of the upper esophageal sphincter (UES), and intraoperatively in guiding the diverticulectomy.

## Case presentation

**Fig. 1:**
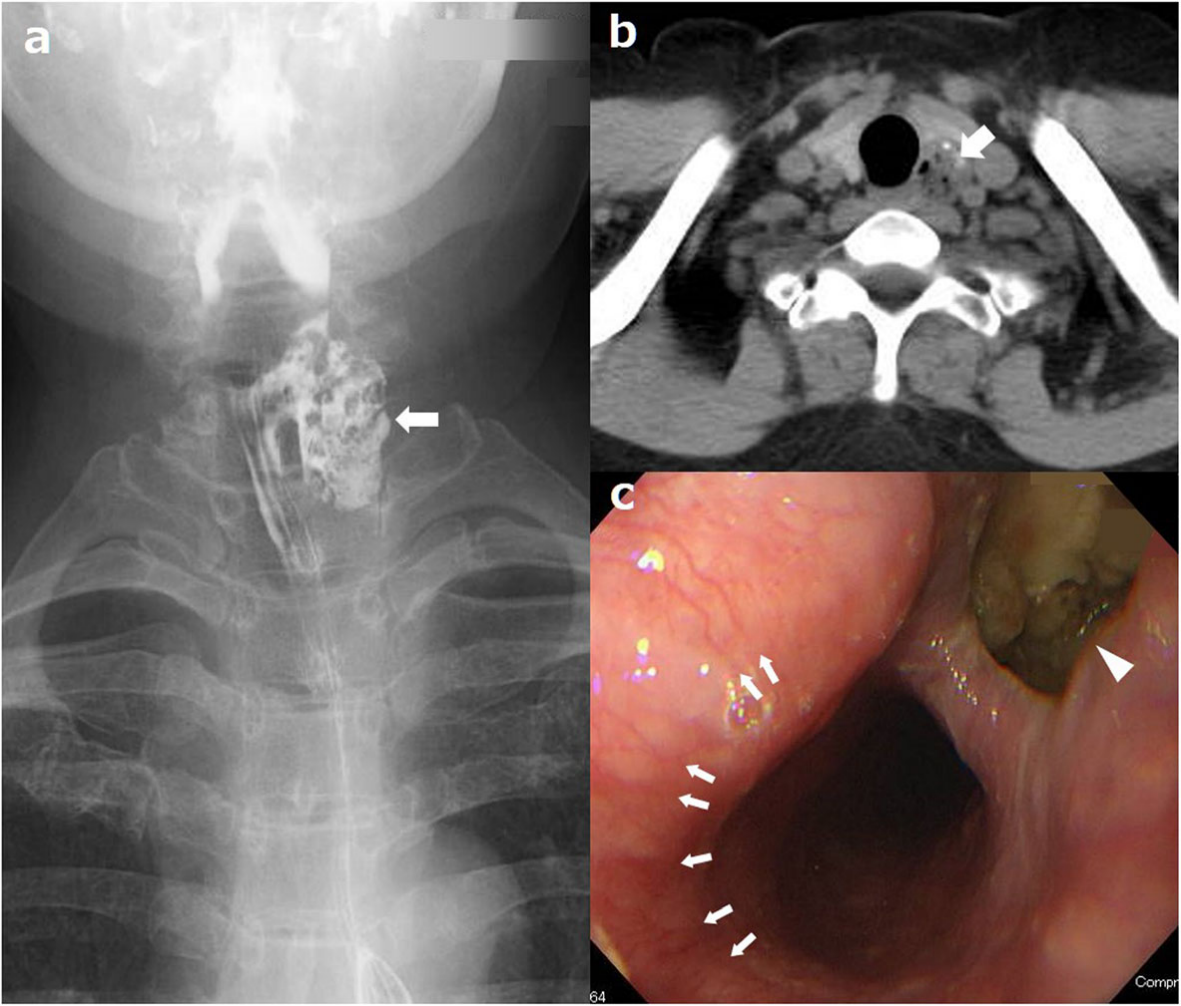
Preoperative findings. **a** Contrast esophagography showing a diverticulum 3 cm in diameter on the left side of the cervix (arrow) but not the cricopharyngeal bar. **b** Cervical computed tomography showing an air-filled diverticulum (1.6 × 1.8 × 3.2 cm) arising from behind the left lobe of the thyroid (arrow). **c** Upper endoscopy showing a diverticulum filled with food residue (arrowhead) just below the palisade vessels corresponding to the level of the UES (arrows)

A 65-year-old woman was referred to our institution with a 5-year history of pharyngeal discomfort and increased coughing. Contrast esophagography revealed a diverticulum measuring 3 cm in diameter on the left side of the cervix (Fig. 1a). Cervical computed tomography showed an air-contained diverticulum measuring 1.6 × 1.8 × 3.2 cm arising behind the left lobe of the thyroid (Fig. 1b). Upper endoscopy confirmed that the diverticulum was filled with food residue just below the palisade vessels, corresponding to the level of the UES (Fig. 1c). High-resolution manometry showed no abnormalities other than an area with slightly high pressure in the cervical esophagus. The diagnosis was KJD arising below the cricopharyngeus muscle, and she underwent simple diverticulectomy without cricopharyngeal and esophageal myotomy.

**Fig. 2:**
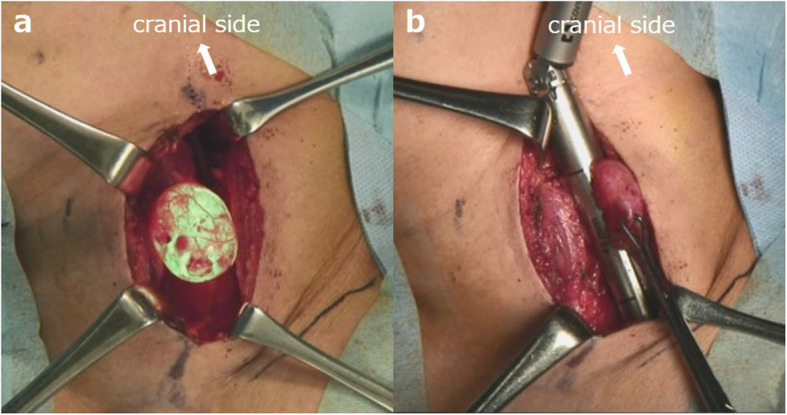
Intraoperative findings. **a** The diverticulum illuminated by the endoscope during surgery was exposed safely. **b** Using an endoscope as a bougie to avoid narrowing of the esophagus, the base of the diverticulum is transected using a linear stapler

An approximately 8-cm oblique incision was made in the left neck, and the anterior cervical muscle and thyroid gland were separated to expose the esophagus and the diverticulum under light from an endoscope (Fig. 2a). The left recurrent laryngeal nerve running on the surface of the diverticulum was identified and preserved. After washing out the food residue inside the diverticulum endoscopically, the diverticulum was confirmed to arise below the palisade vessels and was KJD. Using the endoscope as a bougie to avoid narrowing of the esophagus, the base of the diverticulum was transected using a linear stapler (Fig. 2b). The staple line was embedded and reinforced with absorbable thread, and the operation was completed. The postoperative course was uneventful, and she was discharged on postoperative day 8 with good food intake. At 6 months after surgery, she remains asymptomatic without recurrence of the diverticulum or stenosis at the surgical site.

## Discussion

**Fig. 3:**
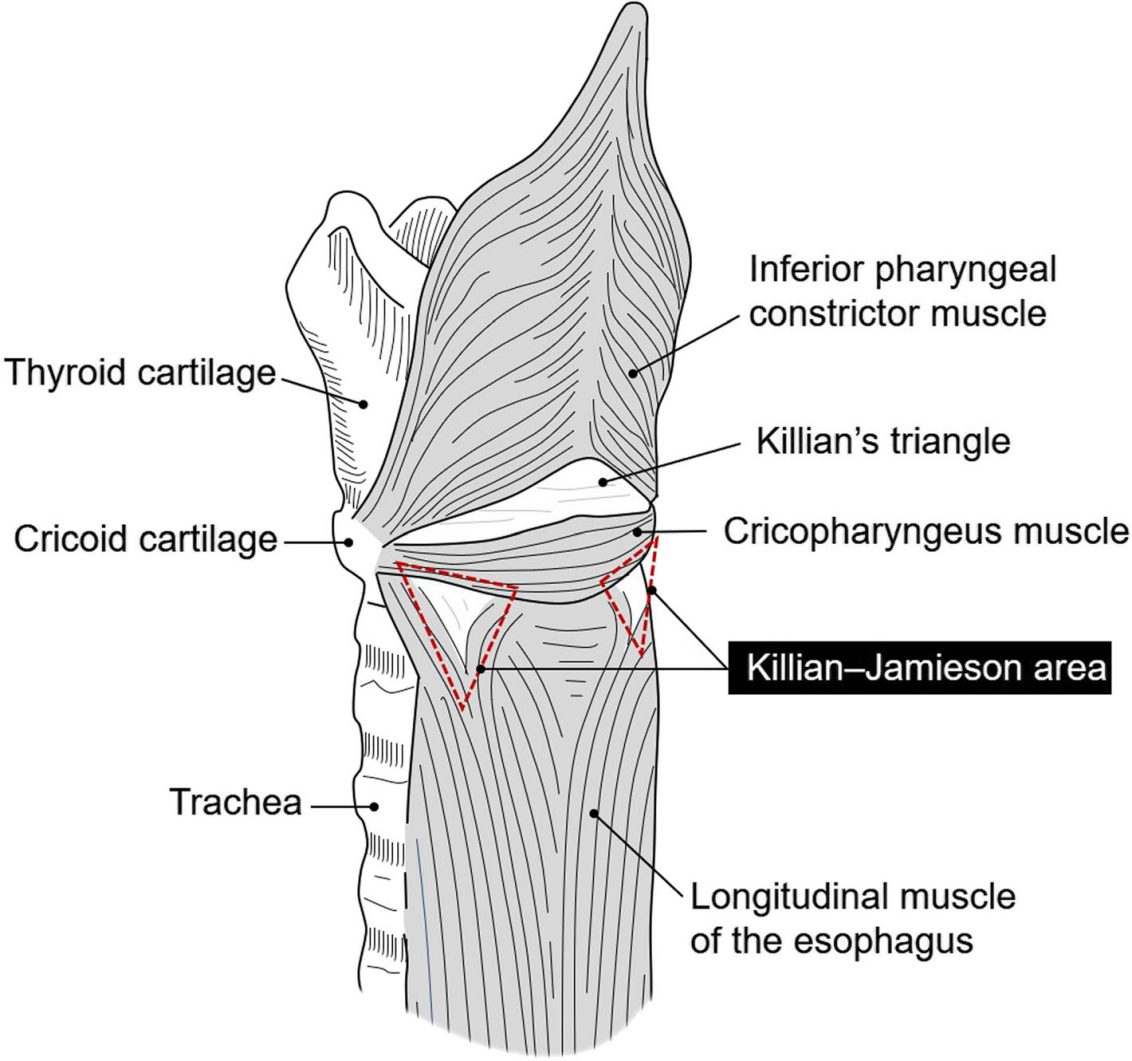
Illustration of the pharyngoesophageal area. ZD arises through a muscular gap in the posterior wall below the inferior pharyngeal constrictor muscle and above the cricopharyngeus muscle, known as Killian’s triangle. KJD arises through a muscular gap in the anterolateral wall of the cervical esophagus just below the cricopharyngeus muscle and superolateral to the longitudinal muscle of the esophagus, known as the Killian–Jamieson area

The pharyngoesophageal diverticulum is classified as either ZD, the most common type, or KJD. ZD arises from a muscular gap in the posterior wall below the inferior pharyngeal constrictor muscle and above the cricopharyngeus muscle, known as Killian’s triangle (Fig. 3) [3]. KJD arises from a muscular gap in the anterolateral wall of the cervical esophagus just below the cricopharyngeus muscle and superolateral to the longitudinal muscle of the esophagus, known as the Killian–Jamieson area (Fig. 3) [3, 5]. First-line treatment for pharyngoesophageal diverticulum is open surgery, which involves diverticulectomy or diverticulopexy followed by cricopharyngeal and esophageal myotomy. Myotomy is mandatory for ZD because its pathogenesis is regarded as insufficient relaxation of the muscles that comprise the UES and high pressure in the hypopharynx [1–3]. Unlike ZD, KJD is not associated with UES dysfunction and does not therefore require myotomy [2, 4]. Differential diagnosis to distinguish between the two types of diverticulum is thus vital in deciding the surgical procedure.

Esophagography is commonly used to distinguish between the two types [3, 6, 7]. ZD is located above the cricopharyngeal bar, which represents constriction of the cricopharyngeus muscle that comprises the UES, whereas KJD is located below the cricopharyngeal bar. In patients with ZD, the cricopharyngeal bar is clearly visible during the swallowing phase due to the problem with the UES opening. In contrast, in patients with KJD, the bar is sometimes invisible during esophagography because there is no associated UES dysfunction [7]. Endoscopically, it is well known that the palisade vessels are observed in the lamina propria corresponding to the UES and the lower esophageal sphincter [8]. The lower palisade vessels can be used to estimate the esophagogastric junction, that is, the muscular boundary of the esophagus and the stomach [9]. Correspondingly, the upper palisade vessels are useful for estimating the level of the UES, helping to distinguish between the two types of diverticulum that prolapse above and below the UES. Thus, upper endoscopy may be another means for differential diagnosis supporting esophagography.

In this case, intraoperative endoscopy was performed under the favorable condition of general anesthesia, and the palisade vessels were more easily and clearly identified than before surgery, which made the diagnosis of KJD more confident. There are some reports of endoscopic diverticulectomy in which a rigid laryngoscope was useful for good visualization of the diverticulum [10, 11]. Intraoperative endoscopy under the larynx deployment using a laryngoscope might provide a clear identification of the palisade vessels for more confident differential diagnosis and also provide new information on the upper edge of the palisade vessels.

## Conclusion

We reported a case of KJD in which upper endoscopy in addition to esophagography was useful in determining the type of diverticulum and the optimal surgical procedure. Specifying the positional relationship between the diverticulum and the palisade vessels that indicate the level of the UES can facilitate the preoperative diagnosis of KJD. Diverticulectomy can be performed intentionally and safely with the aid of intraoperative endoscopy.

## Data Availability

The authors declare that all data in this article are available within the article.

## Abbreviations

KJD: Killian–Jamieson diverticulum
ZD: Zenker’s diverticulum
UES: Upper esophageal sphincter
